# Biophysical aspects of migrasome organelle formation and their diverse cellular functions

**DOI:** 10.1002/bies.202400051

**Published:** 2024-06-23

**Authors:** Raviv Dharan, Raya Sorkin

**Affiliations:** 1 School of Chemistry, Raymond & Beverly Sackler Faculty of Exact Sciences, Tel Aviv University, Tel Aviv, Israel; 2 Center for Physics and Chemistry of Living Systems, Tel Aviv University, Tel Aviv, Israel

**Keywords:** bending rigidity, membrane curvature, membrane tension, migrasomes, tetraspanin

## Abstract

The transient cellular organelles known as migrasomes, which form during cell migration along retraction fibers, have emerged as a crutial factor in various fundamental cellular processes and pathologies. These membrane vesicles originate from local membrane swellings, encapsulate specific cytoplasmic content, and are eventually released to the extracellular environment or taken up by recipient cells. Migrasome biogenesis entails a sequential membrane remodeling process involving a complex interplay between various molecular factors such as tetraspanin proteins, and mechanical properties like membrane tension and bending rigidity. In this review, we summarize recent studies exploring the mechanism of migrasome formation. We emphasize how physical forces, together with molecular factors, shape migrasome biogenesis, and detail the involvement of migrasomes in various cellular processes and pathologies. A comprehensive understanding of the exact mechanism underlying migrasome formation and the identification of key molecules involved hold promise for advancing their therapeutic and diagnostic applications.

## Introduction

**Figure 1:**
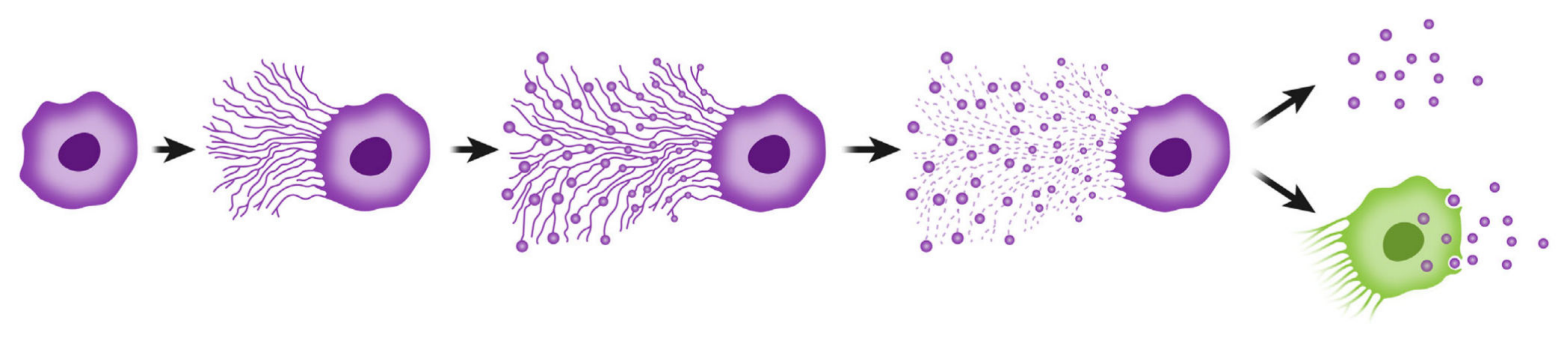
Schematic illustration of migrasome biogenesis. Following cell migration, retraction fibers form, and subsequent membrane swelling along these fibers leads to migrasome formation. While the retraction fibers eventually degrade, migrasomes remain intact. The migrasomes can undergo uptake by other cells or rupture and release their content into the extracellular environment.

Migrasomes are the recently discovered transient cellular organelles formed along retraction fibers, the cylindrical plasma membrane protrusions generated in the course of cell migration.^[1]^ The retraction fibers are branched tubes anchored to the extracellular substrate, measuring tens of microns in length and approximately 100 nm in thickness.^[2]^ Following the formation of retraction fibers, abrupt local membrane swelling leads to the biogenesis of migrasomes. During this process, migrasomes accumulate cytoplasmic content. Ultimately, the retraction fibers undergo degradation and detach from the cells, leaving behind migrasomes. The migrasomes remain intact up to several hours, and eventually rupture and release their content to the environment or alternatively, undergo uptake by recipient cells^[1]^ (Figure 1). These organelles were discovered by the Li Yu lab in 2015, and were first described as pomegranate membrane vesicles that contain smaller vesicles.^[1]^ The term migrasomes was coined because their formation depends on cell migration. While migrasomes had been observed in earlier studies, these studies mainly explored retraction fibers, while the migrasomes have been ignored.^[3,4]^

Even though the study of migrosomes is fairly new and much is yet to be learned about their biogenesis and function, increasing evidence demonstrates that migrasomes are pivotal for a myriad of fundamental cellular processes. For instance, migrasomes encapsulate various cargos, facilitating the transfer of mRNA and proteins between cells, thus functionally modifying the recipient cell.^[5]^ Additionally, migrasomes transport and release damaged mitochondria outside cells, contributing to the maintenance of mitochondrial quality control.^[6]^ It was further demonstrated that migrasomes spatially coordinate organ morphogenesis and promote embryonic angiogenesis in vivo,^[7,8]^ indicating that they are distinguished from the known extracellular vesicles and their functions go beyond those of ordinary signaling vesicles like exosomes.^[9–11]^ In fact, migrasomes are revealed to be imperative cell organelles that become extracellular vesicles once they detach from the retraction fibers. Migrasomes were shown to be involved in a wide range of cellular pathologies and were described as promising therapeutic agents.^[12–14]^ Thus, it is of great importance to understand the physico-chemical mechanism underlying migrasome biogenesis.

It was recently suggested that the interplay between tetraspanins (TSPANs) and membrane tension, bending rigidity, and curvature modulate the process of migrasome generation. TSPANs, a family of 33 known small transmembrane proteins in humans, are extensively involved in membrane remodeling processes and are associated with various cellular functions including cell adhesion,^[15]^ immune signaling,^[16]^ cell fusion,^[17,18]^ viral infection,^[19]^ and cancer metastasis.^[20]^ These proteins interact with themselves and other proteins and lipids, forming domains essential for their diverse functions.^[21,22]^ Notably, 14 TSPANs have been demonstrated to promote migrasome formation.^[23]^ TSPAN functions in general, and in migrasome formation in particular, have been tightly related to the biophysical properties of the membrane, including the membrane tension, curvature and bending rigidity.^[23–25]^ In this review we summarize fundamental studies exploring migrasome formation and functions, emphasizing the biophysical aspects of migrasome biogenesis.

## Migrasome Formation Mechanism

**Figure 2:**
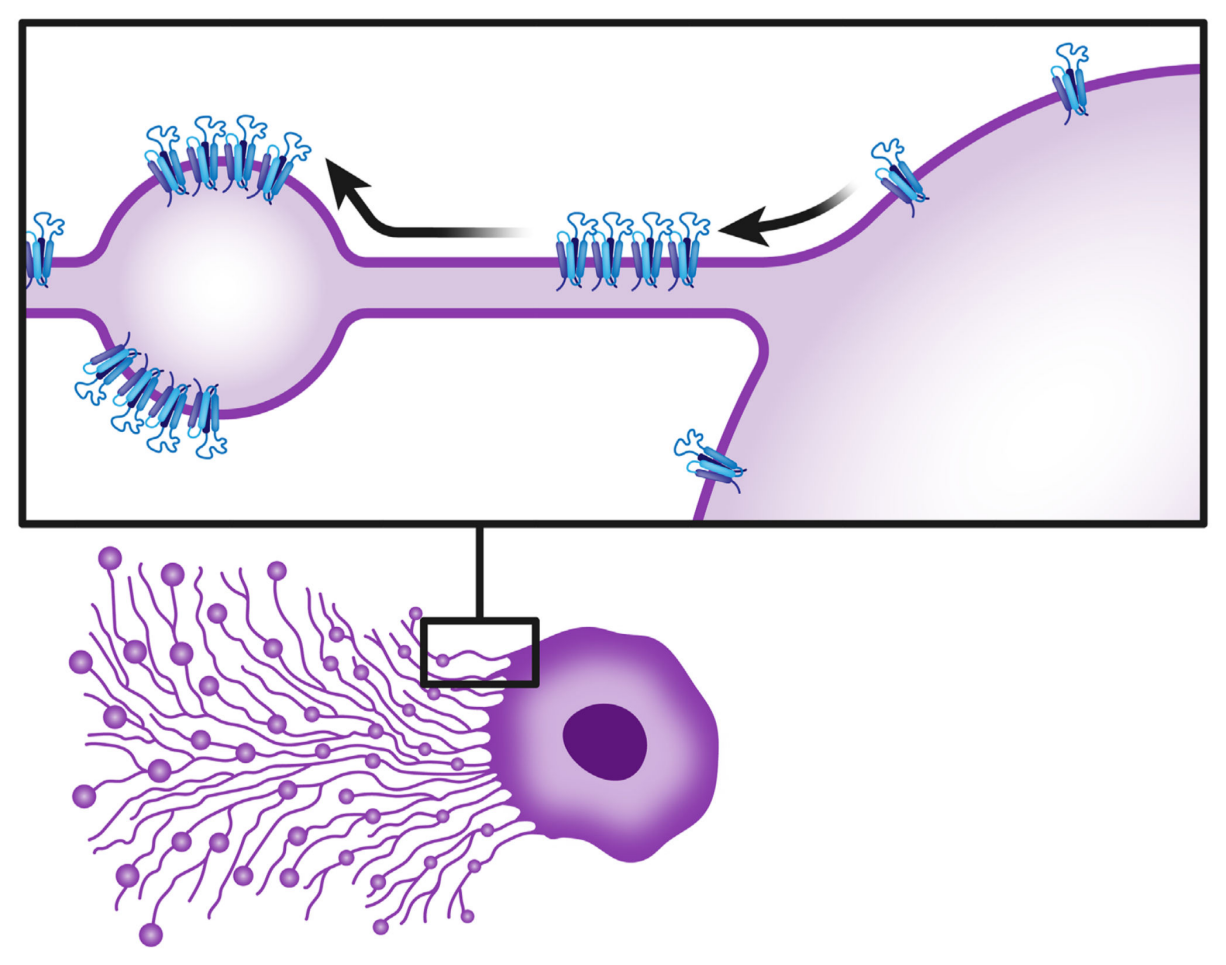
Schematic illustration depicting TSPAN enrichment in retraction fibers and migrasomes. The inset offers a close-up view of migrasomes along a retraction fiber pulled from a cell. Initially, TSPANs are enriched within the highly curved retraction fibers and form TEMs. Next, as migrasomes form, these domains migrate towards the lower curvature of the migrasomes. Illustration based on ref. [25]. TSPAN, tetraspanin.

During the formation of retraction fibers, TSPANs are enriched within these tubular membranes due to their positive intrinsic curvature^[24,26]^ (Figure 2). This enrichment increases the proteins’ probability to interact and form TSPAN-enriched domains (TEMs), which were demonstrated to mediate migrasomes formation.^[23,27]^ These nano/micro domains are physically and functionally distinct from lipid rafts,^[28]^ but share the common attribute of being segregated from other components in the membrane and have similarities in their membrane composition like the enrichment of cholesterol.^[29,30]^ A reconstitution membrane model of giant unilamemlar vesicles containing TSPAN4 and cholesterol revealed that these components alone are sufficient to induce migrasome-like structures.^[23]^ It was further demonstrated that migrasomes are enriched with TEMs, and that TSPANs can increase the membrane bending rigidity and thereby promote migrasome growth (as such domains with increased bending rigidity will favor less curved structures). A recent study provided further insights into the mechanism of migrasome biogenesis, and revealed that migrasomes develop in a two-stage process.^[25]^ In the initial stage, nucleation and early growth of migrasomes begins as small swellings devoid of TSPANs, which are likely driven by membrane mechanical stress such as fluctuations in the membrane tension.^[25]^ The notion that membrane tension increase can lead to migrasome nucleation and initial growth is based on a biomimetic assay emulating migrasome formation. This assay involves pulling a membrane tube from an aspirated giant plasma membrane vesicle (GPMV) containing TSPANs, mimicking a retraction fiber pulled from a cell. A membrane tension jump was used to induce tube pearling instability, leading to the formation of swellings along the tube.^[25,31]^ The ability of membrane tension increase to lead to migrasome nucleation was further substantiated by a live cell imaging assay, where an increase in the intracellular pressure (resulting in increased membrane tension) promoted the formation of migrasome-like structures.^[32,33]^ Other recent studies have revealed further insights into the biomolecules involved in migrasome nucleation. Sphingomyelin synthase 2 (SMS2), ceramide, and sphingomyelin were found to be enriched in migrasomes and important for their biogenesis. The nucleation of migrasomes was suggested to be initiated by the assembly of SMS2 foci, which initially form on the basal membrane at the leading edge of the cell and remain on the retraction fibers during cell migration. These foci become the sites of migrasome formation, where conversion of ceramide to sphingomyelin by SMS2 is suggested to facilitate migrasome growth.^[34]^ Additionally, calcium ions also regulate the initial growth of migrasomes. The calcium-sensor protein synaptotagmin-1 (Syt1) was found to be enriched in migrasomes and essential for their formation. It has been demonstrated that the binding of calcium to Syt1 is a key factor in the process. The recruitment of Syt1 triggers the initial swelling of the retraction fibers, leading to the formation of unstable precursors of migrasomes. These premigrasomes continuously change their shapes or shrink back to the retraction fibers,^[35]^ unless they are stabilized in the next stage of migrasomes biogenesis. The precise mechanisms controlling the assembly of SMS2 foci, as well as the initial swelling triggering by syt1, are yet to be fully elucidated.

In the second stage of migrasome formation, TEMs migrate towards and onto the swellings, stabilizing them and facilitating their growth into fully formed migrasomes (Figure 2). The enrichment of TSPANs in the migrasomes is crucial for their stabilization, as in the absence of TSPANs, the swellings quickly dissipate back into the retraction fibers. The precise mechanism by which TSPANs migrate and stabilize the premigrasome structure is still unclear.

Following their initial formation as small swellings, migrasomes grow into spherical membrane structures of varying sizes, ranging from 0.5 to 3 μm.^[1,27]^ Thus, migrasomes exhibit significantly smaller membrane curvature compared to retraction fibers. The fact that TSPANs first become enriched within the high membrane curvature of retraction fibers and then move into the effectively planar membrane of the migrasomes can be explained as follows; While individual TSPAN molecules possess a high positive intrinsic curvature, the assembly of TSPANs into the higher order domains of TEMs results in a structure with lower intrinsic curvature. Alternatively, the formation of TEMs may increase the bending rigidity of the membrane, leading to preference of low curvature structures. The mechanism of TSPAN enrichment in migrasomes requires further examination, particularly through assays exploring the physical properties of TEMs within the membrane.

In addition to migrasomes, other membrane structures form from retraction fibers. The smallest known membrane vesicles derived from the retraction fibers are termed retractosomes. Retractosomes have been observed in vivo in neutrophils in mice, and in embryonic cells during zebrafish embryonic development.^[36]^ They typically fall within the range of 50−250 nm and are distinct from migrasomes and other extracellular vesicles.^[36]^ The retractosomes form following the breakage of retraction fibers during which the membrane pearls into small connected spheres, resembling beads on a string. They are larger than typical exosomes, and lack their classical markers such as TSG101, CD63, and syntenin-1 protein.^[10]^ Furthermore, although retractosomes and migrasome membranes are enriched with TSPANs and have other similarities in their protein composition, they have distinct membrane composition and cargo. Notably, retractosomes lack cholesterol, a critical component found in migrasomes.^[23,25]^ This observation correlates with the finding that TEMs, which include cholesterol,^[29,37,38]^ are enriched in the low curvature migrasome membranes, whereas individual TSPANs that remain on the highly curved retraction fibers are enriched in the small retractosomes.

## Mechano-Signaling In Migrasome Formation

Along with TSPANs and cholesterol, the crucial factors for migrasomes formation, additional proteins and lipids were found to be important. In particular, proteins associated with cellular adhesion and mechano-signaling are closely associated with migrasome formation. Integrins were found to become enriched in migrasomes, concentrating at their bottom side where they interact with the extracellular matrix.^[39]^ Unlike TSPANs which are found in both migrasomes and the retraction fibers, integrins are exclusively enriched in migrasomes. This suggests their potential role in promoting migrasome substrate adhesion and stabilization, allowing migrasomes to remain intact after the retraction fiber degradation.

Tumor necrosis factor A was also shown to induce formation of retraction fibers in primary human coronary artery endothelial cells, resulting in an increase in cell adhesion and migrasome formation.^[40]^ Cell adhesion was further demonstrated to have a critical role in migrasome formation likely by generating membrane stresses during cell migration. An increase in the concentration of fibronectin led to a higher number of migrasomes per cell, indicating that migrasome formation is influenced by cell adhesion to the substrate.^[41]^ Rho-associated kinase 1 (ROCK1), a serein/threonine protein kinase, was identified as a regulator of migrasome formation through its role in cell adhesion to the extracellular substrate fibronectin.^[41,42]^ These interactions are generating traction forces, which may promote the initial swelling of the membrane during migrasome biogenesis. In addition, inhibition of ROCK1 significantly reduced the number of migrasome generated by podocytes.^[12]^ Another study with peptide-modified substrates demonstrated that, compared to fibronectin, several cell-penetrating (peptide vascular endothelial cadherin (pVEC) and R9) and virus fusion (simian immunodeficiency virus (SIV)) peptides can further enhance cell adhesion levels, resulting in increased cell migration, elon-gation of retraction fibers, and a greater number of migrasomes.^[43]^

Furthermore, the programmed death ligand 1 (PD-L1), which usually functions as an immune checkpoint, has also been found in retraction fibers and migrasomes and was suggested to have a role in the regulation of membrane tension.^[44]^ It was demonstrated that PD-L1 promotes persistent cell migration by accumulating at the rear of migrating cells where it interacts with integrin *β*4 to regulate its association with actin. This association activates the small GTPase RhoA which promotes contractility and thereby increases actomyosin tension.^[44,45,46]^ This process stimulates cell rear retraction and the consequent formation of retraction fibers and migrasomes containing PD-L1 and integrin.^[44]^

Several other proteins, like bifunctional heparan sulfate N-deacetylase/N-sulfotransferase 1 (NDST1), phosphatidylinositol glycan anchor biosynthesis, class K (PIGK), carboxypeptidase Q (CPQ) and epidermal growth factor (EGF) domain-specific O-linked N-acetylglucosaminetransferase (EOGT), are also enriched in migrasomes, but not exosomes, and thus they are distinguished from TSPANs and integrins which are enriched in both of these vesicles. Therefore, these proteins were identified as migrasome markers, and were used to detect migrasomes in human serum.^[47]^ Another identified marker for migrasomes is the pleckstrin homology domain,^[7]^ a protein domain involved in cell signaling and cytoskeletal function.^[48]^ Actin, myosin, and annexin proteins have also been detected in migrasomes,^[13]^ yet experimental data showing their specific functions in migrasome formation is still lacking. Further research is needed to elucidate the precise mechano-signaling pathways in migrasome formation, including the pathways generating membrane mechanical stresses that may lead to initial migrasome swelling.

## Migrasomes In 3-Tube Junctions

**Figure 3:**
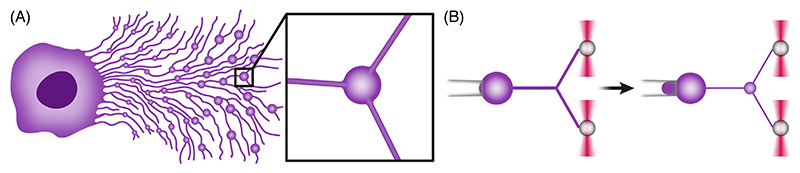
Retraction fibers form 3-tube junctions, the preferred location for migrasome formation. (A) Schematic illustration of a cell with retraction fibers and migrasomes formed during cell migration. The inset offers a close-up view of a migrasome in a 3-tube junction. (B) Schematic illustration of membrane swelling at a 3-tube junction pulled from an aspirated GPMV, formed as a result of an abrupt tension increase. This setup mimics a 3-tube junction formed by cellular retraction fibers. The left panel depicts low aspiration pressure (corresponding to low membrane tension), while the right panel depicts abruptly increased aspiration pressure (corresponding to high membrane tension). GPMV, giant plasma membrane vesicle. Illustration based on ref. [32].

Migrasome formation is strictly dependent on cell migration. The migration of cells can be impersistent and exhibit turns. A recent study demonstrated that the patterns of cell migration affect migrasome formation.^[49]^ For example, cells form fewer migrasomes when making turns due to narrower cell rear ends leading to fewer retraction fibers during turning. In addition, a higher migration speed correlates with an increase in migrasome formation, probably by promoting mechanical stresses that induce membrane bulging. These migration patterns lead to extensively branched retraction fibers with 3-way tubular junctions. The junction, where 3 membrane tubes intersect at 120° angle, was observed to be the preferred location of migrasomes along the retraction fibers (Figure 3A).^[1]^ The preference of migrasome formation at these junctions was recently studied theoretically and experimentally substantiated using live cell and biomimetic assays.^[32]^ Computational analysis showed that membrane bulging at the junction site is more energetically favorable than in the tubular regions. The live cell assay further validated this preferential membrane bulging at junctions, especially following an increase in intracellular pressure resulting from exposure to a hypotonic medium. Similarly, the biomimetic assay of three tubules connected by a 3-way junction pulled from a GPMV, emulating a 3-tube junction pulled from a cell, showed the same preference for junction swelling formation following a tension jump (Figure 3B).^[32]^ A possible biological rationale for migrasome formation at the junction can be to facilitate migrasome loading with luminal and membrane cargo, as the junction is the intersection of three tubes rather than a single tube.

## Migrasomes In Vivo And Their Therapeutical Potential

Migrasomes have been identified in diverse organisms, suggesting their widespread presence and biological significance. As mentioned above, migrasomes have been detected in human serum, however, their origin and function there is still unclear.^[47]^ Further, migrasomes have been observed during zebrafish gastrulation, where they play an essential role during embryonic development and organ morphogenesis by releasing signaling molecules that provide regional biochemical cues for correct cell positioning.^[7]^ Large quantities of migrasomes were formed 5.5 h post fertilization. The migrasomes were mainly located in extracellular pockets between the endoderm and the yolk syncytial layer and were suggested to be generated by mesodermal and endodermal cells.

Furthermore, migrasomes accumulate cellular waste and can facilitate the disposal of damaged organelles.^[6]^ It was demonstrated that migrating cells can shed damaged mitochondria through migrasomes, a process that was termed mitocytosis. The mitocytosis process has been observed in neutrophils and was crucial for maintaining mitochondrial membrane potential and viability in mice. Additionally, a recent study revealed that migrasomes are generated by monocytes in the chorioallantoic membrane during chicken embryonic development. These cells can deposit migrasomes which are enriched with proangiogenic factors, such as vascular endothelial growth factor A and the chemokine CXCL12, that promote embryonic angiogenesis and recruit more monocytes, creating a positive feedback loop for capillary formation in the chorioallantoic membrane of the embryo.^[8]^ These in vivo studies confirmed the biological relevance of migrasomes by showing their presence in several extracellular spaces of different species. Additionally, they provided further support to the existing cell culture data showing that TSPANs play a critical role in migrasome formation. Moreover, in these studies migrasomes were verified to be a source for signaling molecules, like CXCL12, which are critical for various processes, including organ morphogenesis, angiogenesis, and chemotaxis, vital for fundamental life processes like embryo development.

Migrasomes have also been detected in mice urine after their formation by podocytes.^[12]^ It was demonstrated that podocyte injury can severely increase migrasome formation and the levels of migrasome in urine can serve as an indicator for early podocyte injury. Additionally, migrasomes were generated by blood vessel adherent neutrophils during migration in mouse livers.^[50]^ In the same study, the formation of migrasomes within blood vessels as tumor cells migrated was observed in both mice and zebrafish. It was demonstrated that the migrasomes detached from their origin and could circulate in the vessels. Thus, neutrophils and probably other cells may deliver signals and communicate with cells further from their immediate surroundings, indicating that migrasomes can serve as signaling messengers for longdistance cell-cell communication.

Migrasomes are also involved in the pathogenesis of brain injury after cerebral ischemia and were identified as potential therapeutic targets in nueropatholgy.^[13]^ Migrasomes were found in postmortem ischemic brains of humans after stroke. It was further shown that neurons form significantly more migrasomes following a high salt diet which promotes acute ischemic stroke. Moreover, the retraction fibers and migrasomes of metastatic cancer cells have been shown to preferentially interact with nanoparticles.^[14]^ The nanoparticles were demonstrated to strongly bind the retraction fibers and migrasomes and it was suggested that these interactions interfere with the uptake of migrasomes by surrounding cells. Moreover, these interactions inhibited cell migration, and were suggested to retard tumor metastasis in vivo. Another migrasome associated pathology is viral infection. For instance, it has been shown that the chikungunya virus nsP1 viral protein can induce migrasome formation.^[51]^ Additionally, migrasomes have been linked to the severe acute respiratory syndrome coronavirus 2 (SARS-CoV2), where incubating platelets with SARS-CoV2 virions led to the release of various extracellular vesicles including migrasomes.^[52]^ These diverse studies collectively suggest that some pathological conditions trigger the formation of migrasomes and underscore their potential therapeutic and diagnostic use. The migrasomes can serve as indicators for various pathologies and act as carrier vesicles for drug delivery and targeted therapy, showing their versatility in clinical contexts.

## Outlook

Migrasome formation is a complex cellular process which is orchestrated by molecular mechanisms and physical forces. Migrasomes are receiving increasing attention following the fundamental studies exploring their nature. Current understanding of migrasomes indicates that diverse migrating cells utilize these organelles as cellular machinery for a range of functions. The therapeutic potential of migrasomes was demonstrated in vivo by their critical involvement in pathological processes like cerebral stroke and cancer metastasis. Despite the considerable progress in understanding the essential roles of membrane tension, bending rigidity and curvature acting together with membrane proteins like TSPANs, the precise mechanism of migrasome biogenesis remains incompletely understood.

What are the exact membrane stresses and molecular mechanisms that trigger migrasome nucleation and initial growth? Why do TSPANs migrate to the migrasome and how do TSPANs proteins stabilize them? Do different TSPANs have different functions in migrasome formation? Why are there many sizes of migrasomes, and are they effectively different?

To address these important questions and to elucidate the exact nature of migrasomes, new assays should be developed, allowing the investigation of the individual membrane physical factors and vital proteins and lipids participating in migrasome formation. Understanding the critical molecules and physical forces involved in the migrasome shaping can pave the way to further advancements in therapeutic and diagnostic applications of these fascinating organelles.

## Data Availability

Data sharing not applicable to this article as no datasets were gener-ated or analyzed during the current study.
